# Aryl Hydrocarbon Receptor Regulates Muc2 Production Independently of IL-22 during Colitis

**DOI:** 10.3390/ijms25042404

**Published:** 2024-02-18

**Authors:** Archana Saxena, Chandani Mitchell, Raymond Bogdon, Kasie Roark, Kiesha Wilson, Shanieka Staley, Michelle Hailey, Michal Claire Williams, Alex Rutkovsky, Prakash Nagarkatti, Mitzi Nagarkatti, Philip Brandon Busbee

**Affiliations:** Department of Pathology, Microbiology, and Immunology, School of Medicine, University of South Carolina, Columbia, SC 29209, USA; archana.saxena@uscmed.sc.edu (A.S.); chandani.mitchell@uscmed.sc.edu (C.M.); raymond.bogdon@uscmed.sc.edu (R.B.); kasie.roark@uscmed.sc.edu (K.R.); kiesha.wilson@uscmed.sc.edu (K.W.); shanieka.staley@uscmed.sc.edu (S.S.); michelle.hailey@uscmed.sc.edu (M.H.); alex.rutkovsky@uscmed.sc.edu (A.R.); prakash@mailbox.sc.edu (P.N.); mitzi.nagarkatti@uscmed.sc.edu (M.N.)

**Keywords:** inflammatory bowel disease, colitis, aryl hydrocarbon receptor, indole-3-carbinol, mucin, interleukin-22, goblet cell

## Abstract

We previously reported that an aryl hydrocarbon receptor (AhR) ligand, indole-3-carbinol (I3C), was effective at reducing colitis severity through immune cell-mediated interleukin-22 (IL-22) production. Intestinal epithelial cells (IECs) are also involved in regulating colitis, so we investigated their AhR-mediated mechanisms in the current report. A transcriptome analysis of IECs in wildtype (WT) mice revealed that during colitis, I3C regulated select mucin proteins, which could be attributed to goblet cell development. To address this, experiments under in vivo colitis (mice) or in vitro colon organoid conditions were undertaken to determine how select mucin proteins were altered in the absence or presence of AhR in IECs during I3C treatment. Comparing WT to IEC-specific AhR knockout mice (AhR^ΔIEC^), the results showed that AhR expression was essential in IECs for I3C-mediated protection during colitis. AhR-deficiency also impaired mucin protein expression, particularly mucin 2 (Muc2), independently of IL-22. Collectively, this report highlights the important role of AhR in direct regulation of Muc2. These results provide justification for future studies aimed at determining how AhR might regulate select mucins through mechanisms such as direct transcription binding to enhance production.

## 1. Introduction

Inflammatory bowel diseases (IBDs), such as ulcerative colitis (UC) and Crohn’s disease (CD), are a collection of disorders characterized by chronic and dysregulated inflammation in the gastrointestinal tract (GI), particularly the large intestine or colon. The exact etiology of IBDs is currently unknown, but most experts agree four major factors contribute to disease development and progression, which include immune cell dysregulation, genetics, the gut microbiome, and other environmental triggers [1,2]. IBD incidence is increasing worldwide, particularly in industrialized countries [3,4], and this trend correlates with increased healthcare costs associated with the affected patient population [5]. In the US alone, which has estimates as high as 3.6 million adult IBD cases, the annual health care costs associated with this disease increased from USD 6.4 billion to USD 25.4 billion from 1996 to 2016 [6]. Part of the rise in healthcare cost, in addition to increased incidence, can be attributed to the lack of a cure for IBD patients, and standard care options often result in negative side-effects or decreased efficacy after prolonged use, in addition to nonresponding patients [7,8]. With increasing prevalence, costs, and unmet challenges in current treatment options for IBD patients, there remains a high demand for ongoing research meant to better understand the mechanisms associated with IBD and identify therapeutic targets which provide long-lasting beneficial effects without deleterious side-effects.

One potential therapeutic target that continues to gain attention in IBD-related research is the aryl hydrocarbon receptor (AhR). AhR is a cytosolic-bound receptor and basic helix–loop–helix transcription factor activated by receptor-ligand binding which promotes translocation to the nucleus, wherein the AhR complex can subsequently bind to AhR-specific regions of the DNA, known as dioxin response elements (DREs), to regulate gene expression, including genes linked to inflammatory diseases [9]. Previous studies revealed global AhR knockout in mouse models of colitis led to increased colitis disease severity [10,11], and IBD patients were shown to have decreased activation and expression of AhR [12]. We published a report showing indole-3-carbinol (I3C), a natural AhR ligand derived from cruciferous vegetables, was able to reduce colitis severity and disease-associated microbial dysbiosis, attributed predominately through the production of interleukin-22 (IL-22) by innate lymphoid type 3 (ILC3) cells [13]. An interesting finding that deserved further consideration from this report was the fact that I3C treatment led to increased mucin 2 (Muc2) production. Mucin proteins, such as Muc2, are produced by specialized IECs known as goblet cells to maintain the natural barrier and loss of Muc2 promotes spontaneous colitis [14], underlying its important role in maintaining the mucosal barrier. Given the importance of Muc2 production by goblet cells, particularly as it relates to colitis, we wanted to determine, based on our previous findings, if Muc2 production was directly regulated by AhR, the result of indirect effects, such as the alteration of goblet cell development and proliferation capabilities or even consequential response of IECs to increased IL-22 [15,16].

To determine if AhR activation in IECs is directly responsible for increased Muc2 production, we generated conditional AhR knockout mice with deficiency in IECs (AhR^ΔIEC^). Mucin protein expression, along with mature goblet cell and proliferation markers, during in vivo colitis or in vitro organoid cultures with or without I3C treatment, was then evaluated. Importantly, AhR^ΔIEC^ mice no longer respond to I3C treatment during colitis when compared to their wildtype (WT) counterparts, regardless of sex. Interestingly, previously observed protective IL-22 production derived from immune cells was maintained in AhR^ΔIEC^ mice despite no longer showing protection against colitis after treatment with I3C. Most notably, AhR^ΔIEC^ mice showed impairment of Muc2 at steady-state conditions and were unable to restore Muc2 after I3C treatment in colitis, while there were no significant differential effects on goblet cells or proliferation markers in IECs between AhR^ΔIEC^ and WT. IL-22 supplementation of organoids derived from AhR^ΔIEC^ and WT mice did not impact Muc2 production but did affect goblet cell development. Collectively, these results, substantiated by the presence of AhR binding sites in the Muc2 gene promoter region, provide strong evidence that Muc2, a key component in maintaining gut homeostasis, is directly regulated by AhR independently of goblet cell development or cellular response signaling by IL-22.

## 2. Results

### 2.1. Gene Expression Analysis of Colonic Epithelial Cells (CECs) during I3C-Mediated Protection in the 2,4,6-Trinitrobenzene Sulfonic Acid (TNBS) Colitis Model

**Figure 1:**
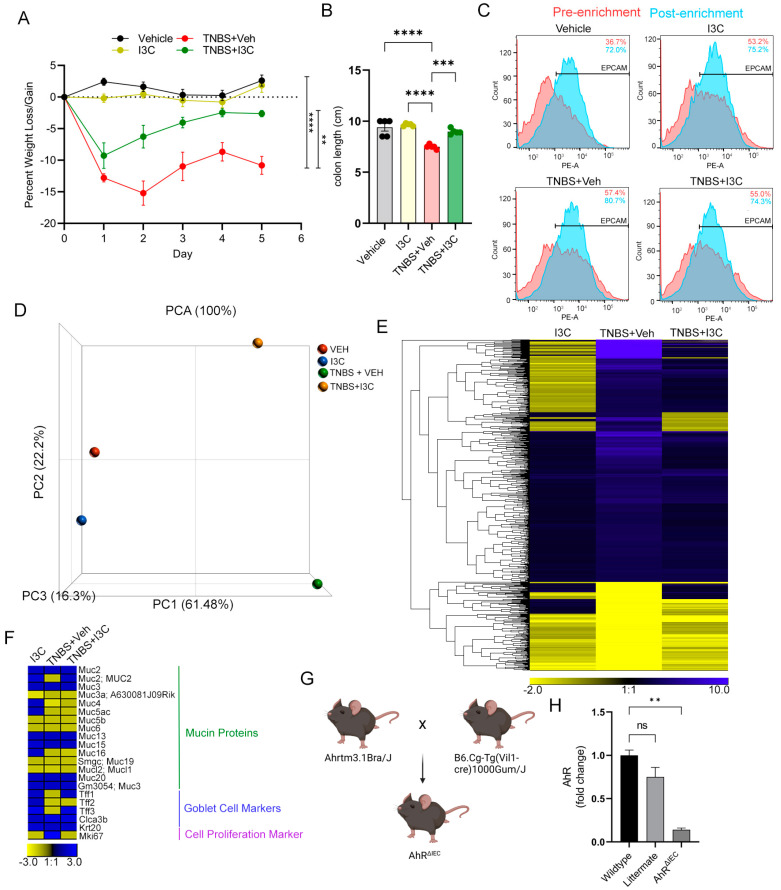
Treatment with I3C reduces TNBS-induced colitis severity and regulates CEC-specific regulatory genes. (**A**) Weight loss data. (**B**) Colon length data. (**C**) Representative flow cytometry plots of pre- and post-selected EPCAM+-enriched colonocytes. (**D**) PCA plot depicting experimental groups for transcriptome analysis. (**E**) Heatmap depicting expressing fold changes compared to Vehicle control of549 differentially expressed coding genes (noncoding removed) with at least a 2-fold change compared to control. (**F**) Heatmap depicting differentially regulated mucin proteins, mature goblet cell markers (Tff1-3, Clca3b), pan-epithelial cell differentiation marker (Krt20) and proliferation marker (Mki67 or mouse ki67). (**G**) Visual diagram illustrating the generation of conditional IEC-specific AhR knockout mice (AhR^ΔIEC^). (**H**) Bar plot depicting PCR results of AhR transcript expression levels between WT, LM, and AhR^ΔIEC^ samples from EPCAM-enriched colonocytes. Error bars equal the standard error mean (SEM). For bar plots, significance was determined using one-way ANOVA and Tukey’s multiple comparisons test. For weight data over time, two-way ANOVA and Dunnett’s multiple comparisons test was used to determine significant (ns, not significant, ** *p* < 0.01, *** *p* < 0.005, **** *p* < 0.001).

We repeated and replicated studies showing that I3C treatment of TNBS-induced colitis in female BALB/cJ mice reduced disease severity. As expected, TNBS colitis mice showed significant loss in body weight and colon length shortening as compared to vehicle controls, but colitis mice treated with I3C (TNBS + I3C) significantly reduced colitis-associated weight loss (Figure 1A) and colon shortening (Figure 1B). To evaluate IEC responses specifically during TNBS colitis and treatment with I3C, CECs were enriched from experimental groups (Vehicle, I3C, TNBS + Veh, and TNBS + I3C), and enrichment purity was assessed by flow cytometry for EPCAM-positive selection, which was found to be between 72 and 80% (Figure 1C). Using enriched CECs, transcriptome microarray analysis was performed to evaluate gene expression patterns after I3C treatment during TNBS colitis in pooled samples from each experimental group. The Partek-generated principal coordinate analysis (PCA) plot showed appropriate spatial clustering of the experimental groups (Figure 1D). A total of 26,596 genes showed up after microarray data analysis using TAC analysis software version 4.01.36, and after applying a filter for only identified coding genes with a ±2-fold change compared to control (Vehicle), 549 coding genes were identified and subjected to ward linkage hierarchical clustering, as depicted in the heatmap (Figure 1E).

Next, attention was focused on significantly altered mucin proteins, mature goblet cell markers (trefoil factors 1–3, Tff1-3; chloride channel accessory 3b, Clca3b), a pan-intestinal epithelial differentiation marker (keratin 20, Krt20), and a cellular proliferation marker (mouse antigen kiel 76, Mki67) among experimental groups (I3C, TNBS + Veh, TNBS + I3C) compared to control. As illustrated in the heatmap (Figure 1F), there were three genes that were significantly downregulated by colitis induction (TNBS + Veh) and restored after disease mice were treated with I3C (TNBS + I3C), which included one mucin protein (Muc2) and two goblet cell markers (Tff1 and Tff3). However, whether Muc2 and/or goblet cell development were directly regulated by AhR or a byproduct some indirect mechanism after AhR activation, such as increased IL-22 production by immune cells as previously reported, was not clear. To address this, conditional AhR-gene knockout mice were generated on the C57BL/6 background using the cre-flox method, with specific AhR knockout in IECs (AhR^ΔIEC^), as illustrated in Figure 1G. Specific AhR knockout in IECs was confirmed by isolating enriched CECs from WT, littermate (LM), and AhR^ΔIEC^ mice and evaluating AhR expression by PCR (Figure 1H). LM mice induced with colitis and treated with I3C showed a statistically significant reduction in weight loss, colon shortening, and overall colonic damage, which effects were lost in AhR^ΔIEC^ mice (Supplemental Figure S1). Depletion of AhR in IECs also did not impact the ability of I3C to enhance the production of IL-22 by ILC3s during colitis (Supplemental Figure S2). Subsequent in vivo and in vitro experiments involving AhR^ΔIEC^ mice to evaluate effects in IECs during colitis and treatment with I3C were performed using WT C57BL/6J mice as controls since this was their parent background.

### 2.2. AhR Deficiency in IECs Leads to Loss of Protective Effects of I3C in the DSS-Induced Colitis Model despite Restoring IL-22 Production in the Colon

**Figure 2:**
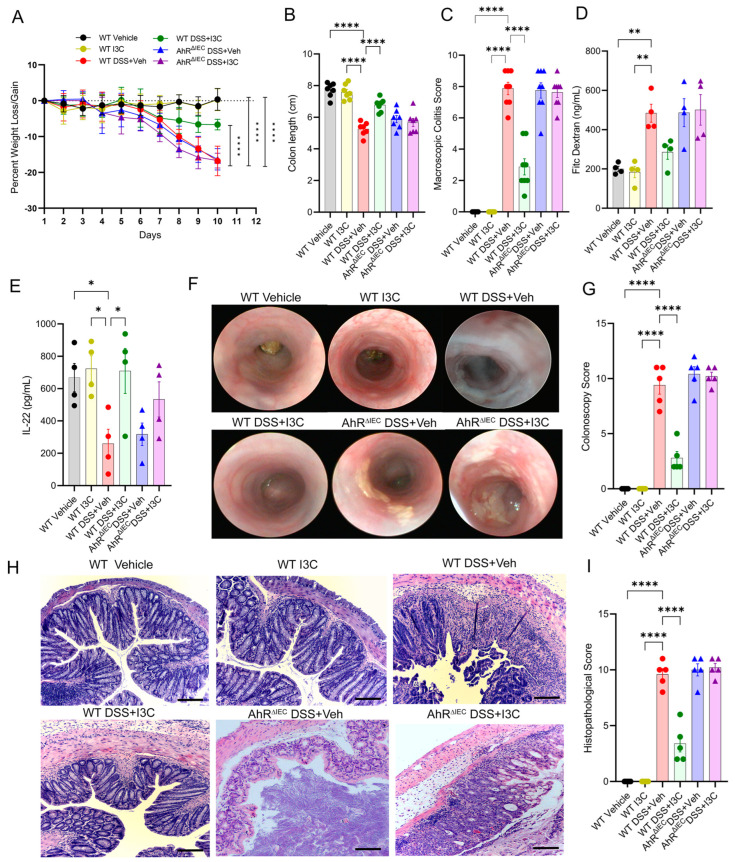
AhR expression in IECs is essential for I3C-mediated protection against DSS-induced colitis in female C57BL/6 mice. (**A**) Weight loss data. (**B**) Colon length data. (**C**) Macroscopic score data. (**D**) FITC-dextran results. (**E**) Bar plot depicting IL-22 ELISA results. (**F**) Representative colonoscopy images taken on day 9 of DSS colitis from experimental groups. (**G**) Bar graph depicting colonoscopy scores. (**H**) Representative H&E images; scale bar (black line) = 50 µm. (**I**) Bar graph depicting histopathological scores from experimental mice (*n* = 5 per group). For bar plots, significance was determined using one-way ANOVA and Tukey’s multiple comparisons test. For weight data over time, two-way ANOVA and Dunnett’s multiple comparisons test was used to determine significance (* *p* < 0.05, ** *p* < 0.01, **** *p* < 0.001).

Female WT or AhR^ΔIEC^ mice were induced with dextran sodium sulfate (DSS) colitis with or without I3C treatment. WT disease controls (WT DSS + Veh) showed significant weight loss over time, which was expectedly restored after treatment with I3C (Figure 2A). However, AhR^ΔIEC^ mice induced with colitis and treated with I3C failed to reverse disease-induced weight loss. In addition, AhR^ΔIEC^ disease mice treated with I3C (AhR^ΔIEC^DSS + I3C) were not able to reverse colitis-induced shortening of the colon (Figure 2B) or reduce overall macroscopic disease scores (Figure 2C), unlike their WT counterparts. Also, while WT colitis mice treated with I3C were able to reduce increased gut permeability, AhR^ΔIEC^ colitis mice treated with I3C failed to prevent “leaky gut” (Figure 2D). In addition, ELISA data for both WT DSS and AhR^ΔIEC^ DSS mice treated with I3C increased the level of IL-22 in the culture supernatant of colon explants (Figure 2E). A colonoscopy evaluation showed AhR^ΔIEC^ mice had marked ulceration and tissue damage during colitis even after treatment with I3C, while WT colitis mice treated with I3C had significant improvement in colonoscopy scores (Figure 2F,G). Lastly, a histological analysis validated the colonoscopy results as there was significant destruction of the colon architecture (e.g., loss of crypts and goblet cells) and evidence of immune cell infiltration in WT and AhR^ΔIEC^ DSS mice, but only WT disease mice had improved histological scores after treatment with I3C (Figure 2H,I).

**Figure 3:**
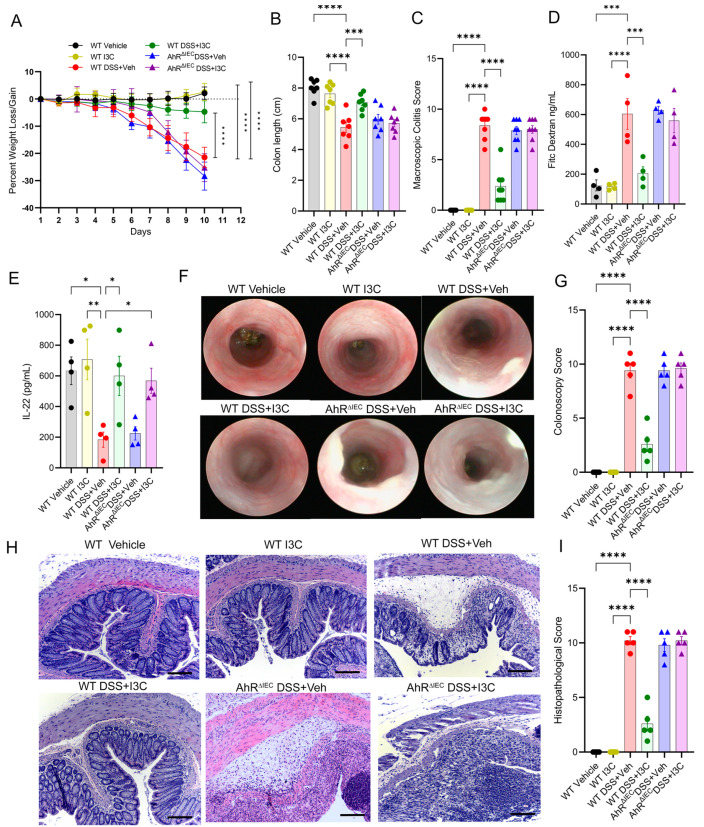
AhR expression in IECs is essential for I3C-mediated protection against DSS-induced colitis in male C57BL/6 mice. (**A**) Weight data. (**B**) Colon length data. (**C**) Macroscopic scoring data. (**D**) FITC-dextran results. (**E**) Bar plot depicting IL-22 ELISA results. (**F**) Representative colonoscopy images taken on day 9 of DSS colitis from experimental groups. (**G**) Bar graph depicting colonoscopy scores. (**H**) Representative H&E stains of colon; scale bar (black line) = 50 µm. (**I**) Bar graph depicting histopathological scores. For bar plots, significance was determined using one-way ANOVA and Tukey’s multiple comparisons test. For weight data over time, two-way ANOVA and Dunnett’s multiple comparisons test was used to determine significance (* *p* < 0.05, ** *p* < 0.01, *** *p* < 0.005, **** *p* < 0.001).

Similar studies in male WT and AhR^ΔIEC^ mice with DSS colitis and I3C treatment were also performed. As seen in females, I3C protects WT mice from DSS-induced injury but not AhR^ΔIEC^ mice, as evident by improvements in body weight (Figure 3A), colon length (Figure 3B), and macroscopic score (Figure 3C). Male WT disease mice treated with I3C also had decreased evidence of gut permeability, while AhR^ΔIEC^ counterparts did not (Figure 3D). In contrast, as was seen in females, IL-22 levels were increased in both WT DSS and AhR^ΔIEC^ DSS male mice after I3C treatment (Figure 3E). Colonoscopy and histological analysis also showed reduced signs of colon tissue damage after I3C treatment in male WT DSS mice but not AhR^ΔIEC^ DSS mice (Figure 3F–I), as was observed in females. These results showed that AhR was essential in IECs to mediate I3C-induced protective effects in the DSS model, regardless of sex, and most interestingly, this was despite retaining the ability to increase or at least restore production of IL-22. How this loss of protection due to AhR deficiency in IECs occurs during I3C treatment was next investigated.

### 2.3. Deficiency of AhR in IECs In Vivo Impairs the Ability of I3C to Increase Mucus Production but Not Goblet Cell Development during Colitis

**Figure 4:**
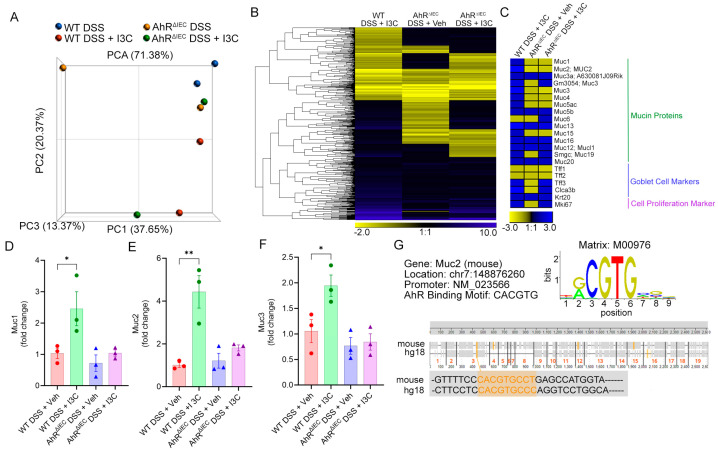
Deficiency of AhR in IECs reduces the ability of I3C to regulate CEC-specific regulatory genes, particularly mucins. (**A**) PCA plot depicting transcriptome results from experimental groups. (**B**) Heatmap depicting fold changes in differentially expressed coding genes (noncoding removed) with at least a 2-fold change compared to control (**C**) Heatmap depicting differentially regulated mucin proteins, mature goblet cell markers (Tff1-3, Clca3b), pan-epithelial cell differentiation marker (Krt20), and proliferation marker (Mki67 or mouse ki67). For validation of microarray data, PCR was performed from colonocytes isolated from experimental groups using primers for (**D**) Muc1, (**E**) Muc2, and (**F**) Muc 3. (**G**) ConTra v3 online browser-generated data depicting AhR binding sites (orange lines) within Muc2 promoter region of mouse (NM_023566) and human (hg18; NM_002457). Error bars equal the standard error mean (SEM). For bar plots, significance was determined using one-way ANOVA and Tukey’s multiple comparisons test. (* *p* < 0.01, ** *p* < 0.01).

As with the TNBS model with I3C treatment, transcriptome array analysis was performed on enriched CECs from WT and AhR^ΔIEC^ in the DSS model. PCA analysis showed appropriate group clustering, with variability observed in the AhR^ΔIEC^ groups (Figure 4A). Identified coding genes with at least a 2-fold change when compared to control (WT DSS) were filtered out, which resulted in a total of 605 coding genes as depicted in the heatmap (Figure 4B). Focus was then applied to significantly altered mucin genes, along with markers of mature goblet cells and proliferation (Figure 2C). Whereas Muc2 was the only mucin significantly restored after I3C treatment in the TNBS model-derived CECs, several mucins increased in WT disease mice after I3C treatment, but this effect was negated in AhR^ΔIEC^ counterparts, which included Muc2. Interestingly, while I3C treated colitis mice (WT DSS + I3C) increased markers of goblet cells (Tff3 and Clca3b) and cellular proliferation (Mki67), this increased expression was not altered in the AhR^ΔIEC^ phenotype. Select mucins from in vivo experimental WT and AhR^ΔIEC^ colitis mice treated with or without I3C were validated in CECs, which included Muc1 (Figure 4D), Muc2 (Figure 4E), and Muc3 (Figure 4F). PCR validation studies aligned with the microarray results, which showed I3C treatment during colitis resulted in increased mucin protein production, but this effect was lost in AhR^ΔIEC^ colitis mice after treatment. Based on this evidence that Muc2 was dependent on AhR in IECs, in silico tools (ConTra v3) were used to identify any possible AhR binding sites (or DREs) with the conserved CACGTG motif in or nearer the promoter region of the mucin gene. Based on criteria detailed in Materials and Methods, there were a total of 5 DREs identified within the mouse Muc2 promoter region, one of which was a shared conserved region also present in the human Muc2 gene (Figure 4G).

### 2.4. AhR Deficiency in Colon-Derived Organoids Results in Impaired Muc2 Production Independently of Goblet Cell Development

**Figure 5:**
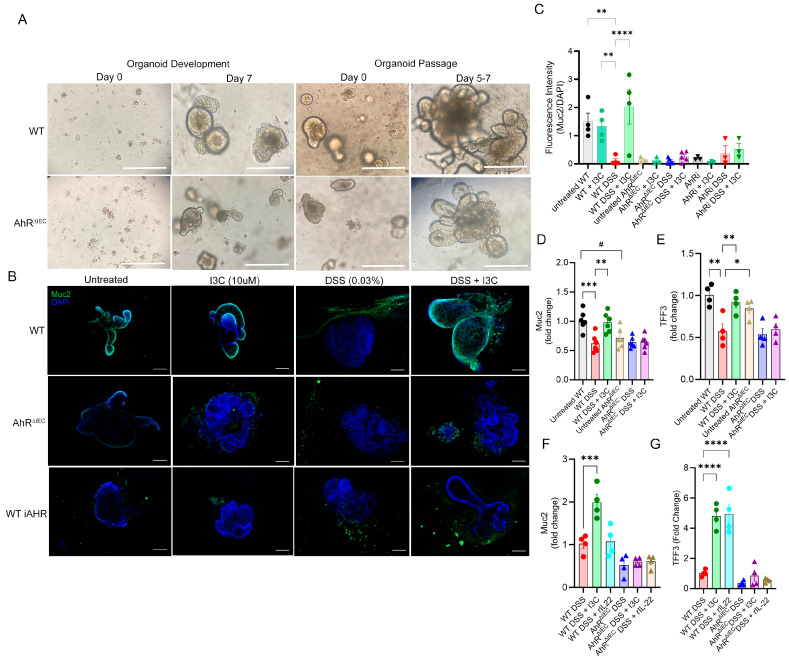
Colon organoids from AhR^ΔIEC^ have impaired ability to produce Muc2 independently, or partly independently, of goblet cell formation/proliferation or IL-22. (**A**) Representative brightfield microscopy images taken during organoid development (Day 0–7) and organoid passaging (Day 0 to Days 5–7); Scale bar (white line) = 50 µm. (**B**) Representative confocal images of colon organoids from WT or AhR^ΔIEC^ mice under detailed experimental conditions; scale bar = 100 µm. (**C**) Bar graph depicting fluorescent intensity of Muc2 and DAPI stains and expressed as a ratio of Muc2/DAPI. (**D**) Bar plot depicting PCR data for Muc2. (**E**) Bar plot depicting PCR data of Tff3. (**F**) Bar plot depicting PCR data for Muc2. (**G**) Bar plot depicting PCR data for Tff3. Error bars equal the standard error mean (SEM). For bar plots, significance was determined using one-way ANOVA and Tukey’s multiple comparisons test using untreated WT at control (* *p* < 0.01, ** *p* < 0.01, *** *p* < 0.005, **** *p* < 0.001). Standard *t*-test was used to compare untreated WT to untreated AhR^ΔIEC^ (# *p* < 0.05).

To eliminate any possible confounding variables that could be associated with other non-IEC AhR-expressing cells having the lower affinity *AhR^d^* allele compared to control WT (higher affinity *AhR^b^* allele), an in vitro system using colon-derived organoids was developed to test more direct response of WT and AhR^ΔIEC^ CECs to I3C, cellular injury, and exogenous IL-22, particularly as it relates to Muc2 and goblet cell development. Images were taken of these developing and passaged organoids using light microscopy, and no obvious differences in the morphology between organoids derived from WT or AhR^ΔIEC^ sources were noted (Figure 5A). After passaging, WT- or AhR^ΔIEC^-derived organoids were under untreated conditions, treated with an in vitro dose of I3C (10 µM), exposed to a low percent of DSS (0.03%) to replicate DSS colitis conditions, or exposed to low DSS concentration with simultaneous treatment with I3C. As shown in Figure 5B, even under untreated conditions, AhR^ΔIEC^-derived organoids had profoundly less Muc2 compared to WT. WT-derived organoids had decreased presence of Muc2 after exposure to DSS, but this was restored in the presence of I3C. As AhR^ΔIEC^-derived organoid cultures had evidence of severe Muc2 production impairment under all experimental conditions, it was thought perhaps these cells had some innate defect in developing goblet cells. To address this, WT-derived organoids were allowed to develop normally and then exposed to the same previous experimental conditions, with the addition of a specific AhR inhibitor (iAhR), CH223191. WT organoid cultures treated with the iAhR were similar to AhR^ΔIEC^-derived organoids, specifically showing impaired AhR led to a significant reduction in Muc2 expression and I3C treatment failed to restore this impairment (Figure 5B,C).

Lastly, PCR validation for genes of interest linked to Muc2 and goblet cell development were evaluated from organoid cultures. The results showed exposure of WT organoids to DSS led to significant decreases in Muc2, which was restored in I3C-treated cultures (Figure 5D), as was evident from IF staining. However, AhR^ΔIEC^ organoids had significantly lower Muc2 when compared to WT, even under untreated conditions, and this low Muc2 expression remained unchanged even after DSS exposure or treatment with I3C. Next, Tff3 was selected as a marker of goblet cell development since Tff3 was found to be significantly changed under in vivo TNBS and DSS conditions, as well as being restored after treatment with I3C. Noticeably, unlike with Muc2, there was no significant difference in Tff3 under untreated or steady-state conditions between WT or AhR^ΔIEC^ organoids (Figure 5E), suggesting AhR does not significantly impact goblet cell formation. DSS exposed WT organoids had decreased expression of Tff3, which was restored after treatment with I3C. Interestingly, Tff3 from AhR^ΔIEC^ cultures were not noticeably restored after DSS exposure and treatment with I3C, which seems to contradict what was seen in under similar in vivo conditions. Lastly, exogenous recombinant IL-22, linked to AhR activation of immune cells, was added to organoid cultures to compare with I3C treatment, in an effort to better understand the potential contribution of this cytokine to either Muc2 or goblet cell development. The results showed Muc2 was not altered by IL-22 supplementation in either WT- or AhR^ΔIEC^ DSS-injured organoids, whereas I3C treatment increased Muc2 in DSS-exposed WT organoids, but this effect was lost with AhR deficiency in IECs (Figure 5F). IL-22 supplementation did increase Tff3 in WT-derived organoids exposed to DSS, as did I3C treatment (Figure 5G). AhR^ΔIEC^ organoids exposed to DSS had no significant differences in Tff3 whether they were treated with I3C or IL-22.

## 3. Discussion

While there have been advancements in standard care for IBD patients over the years, current treatments with antibiotics (ciprofloxacin, metronidazole), aminosalicylates (5-ASAs), corticosteroids (prednisone, hydrocortisone, budesonide), immunomodulators (azathioprine, cyclosporine, methotrexate), and biologics (anti-TNF, anti-integrins) all have potential negative side-effects associated with them, in addition to non-responding patients in some cases. For example, even with first-line of defense, biologics such as anti-tumor necrosis factors (TNFs), it is estimated that 10–40% of IBD patients do not respond to primary treatment [17,18] and almost 50% will stop responding over time [19,20]. Prolonged antibiotic use is not only associated with antibiotic resistance in IBD patients [21] and increased susceptibility for *Clostridium difficile* infection (CDI) [22], but is also itself a potential risk factor for developing IBD [23,24]. Use of broad-based immunosuppressants like corticosteroids are associated with several and even severe adverse events such as increased susceptibility to secondary infections, impaired glucose tolerance, acceleration of atherosclerosis, and increased bone density loss and fracture [25,26,27,28]. These factors present significant unmet challenges in the treatment of IBD patients and highlight the growing need to research and identify more effective therapeutic targets and approaches.

We previously published I3C, a naturally derived AhR ligand and molecule found in cruciferous vegetables, was effective at reducing disease severity in animal models of colitis [13], which was similarly reported by others [29,30,31,32,33]. In one of the earliest reports of this by Li et al., the researchers established that I3C-mediated protective effects were dependent on AhR since global knockout of AhR in mice prevented I3C from reducing disease severity in the DSS model [34]. The current report takes this a step further by illustrating that cell-specific expression of AhR in IECs plays a prominent role in the reduction in colitis severity during I3C treatment. This finding is supported by previous research showing AhR silencing in an epithelial cell line (NCM460) prevented I3C from ameliorating IEC-specific necroptosis and inflammation mediators [29]. More recently, Nolan et al. reported AhR knockout in IECs worsened experimental necrotizing enterocolitis (NEC), which I3C treatment was shown to attenuate [35]. In a report from Peng et al., some of the regulatory mechanisms attributed to AhR specifically in IECs included inhibiting activation of receptor-interacting protein kinase 1 (RIPK1) and formation of the necrosome, suppression of the nuclear factor kappa B (NF-κB) activation pathway, and suppressing inflammatory signaling cytokines such as tumor necrosis factor alpha (TNF-α), IL-1β, IL-6, and IL-8 [29]. Similarly to findings in the current report, Lin et al. concluded AhR activation in the DSS model of colitis increased Muc2 and goblet cell differentiation, which was attributed to the AhR-pErk1/2 signaling pathway [36]. This somewhat contrasts with our own findings, however, as the results revealed herein show AhR regulated Muc2, and this was independent of goblet cell formation. These somewhat conflicting results could be attributed to a few different factors in the studies, including the use of different AhR activating ligands, as well as how mature goblet cells were defined in the respective studies. Researchers in the Lin et al. report defined goblet cells by Muc2 and notch intracellular domain (NICD) protein expression, whereas based on transcriptome results and updated reports, we identified proteins such as Tffs and Clca3b as mature goblet cell markers, similarly to a report by Shah et al. [37]. In fact, findings from Shah et al. aligned with some of the results in the current report. In practical terms, some of the results obtained in the current analysis were consistent with those by Shah et al. In this respect, Shah et al. demonstrated that AhR “was dispensable” in goblet cell growth and differentiation under steady-state circumstances. According to the authors’ study findings, mice that were produced using the same breeding approach as described here and that had AhR depletion in IECs specifically exhibited comparable numbers of goblet cells to those of their wild-type counterparts.

Results in the current report found that AhR knockout in IECs did not appear to affect goblet cell development, but instead, this was regulated more by IL-22, a concept supported in other reports [38,39,40]. Interestingly, Shah et al. found while AhR deficiency did not impair goblet cell development under steady-state conditions, colonic epithelial differentiation in AhR-depleted IECs from in vivo and colon organoid studies was impaired after induction of injury, including goblet cells [37]. Perhaps this is why in the current study there was some discrepancy between in vivo and in vitro colon organoid results in which I3C was able to restore goblet cell markers (Tff3) in vivo after I3C treatment in colitis-induced AhR^ΔIEC^ mice, but not AhR^ΔIEC^-derived organoids. It is possible that AhR deficiency might affect the expression of the IL-22 receptor in IECs/goblet cells, which could impair IL-22 signaling and impact intestinal cellular differentiation. I3C could also be mediating effects through other receptors and not just AhR. Lastly, some of the partial rescue effects observed in the in vivo experiments could be due to incomplete cre expression in the IECs of B6.Cg-Tg(Vil1-cre)1000 Gum/J mice, which was noted in the original publication from Gumucio et al. [41].

Unique to this report is the finding that AhR in IECs regulated Muc2 specifically and independently of effects on goblet cell development and proliferation, which was confirmed using in vivo and colon-derived organoids from AhR^ΔIEC^ mice. It is well established that regulating Muc2 is paramount when it comes to colitis, as Muc2 deletion leads to development of spontaneous colitis in mice [14], which is not seen in mice deficient in other mucins, such as Muc1 or Muc13 [42]. In addition, the important and critical role of Muc2 in the GI tract was illustrated by Bergstrom et al. in a report showing complete loss of mucus staining in Muc2-deficient mice after *C. rodentium* infection, which suggested no other secretory mucin proteins were able to compensate for this loss [43]. However, it is worth noting that there is heterogeneous aberrant expression of different mucins (secretory and transmembrane) in the IBD patient population, making it difficult to ascertain each of these proteins importance during disease states [44]. Another significant finding in the current report was that AhR in IECs was essential in I3C-mediated protection against DSS-induced colitis, which was found to be the case in both male and female mice.

Some limitations in the current report can be addressed in future studies by including an expansion of the control mice population and further investigating the established sex differences that could play a role in AhR activation by I3C during colitis conditions. The current report predominantly used WT C57BL/6J mice as controls since the Ahr^tm3.1Bra^/J mice were created with a floxed AhR gene originating from a mouse strain having the weaker affinity binding *AhR^d^* allele. These mice were later bred and commercialized into C57BL/6 mice, a strain containing the higher affinity *AhR^b^* allele. Thus, some of the reported in vivo results comparing WT to AhR^ΔIEC^ could be due to these mismatched AhR allele differences. However, in the current report we attempted to reduce the potential of confounding factors such as other cell types expressing the weaker affinity AhR allele by developing an organoid system which ultimately negated the contribution of other cells involved in I3C/AhR regulation of Muc2 in IECs. In addition, current ongoing studies with conditional AhR knockout mice will include relevant data from both LMs and WT mice. Also, an initial transcriptome analysis presented herein with the TNBS model of colitis used only female mice, as a previous report from Benson et al. noted that male mice induced with TNBS colitis did not respond to I3C, though females did [33]. In the current report, we included both male and female mice when inducing DSS colitis and treating with I3C to determine if there were any significant differences in treatment response, but both sexes appeared to be equally responsive to I3C. It is worth noting male C57BL/6 mice appeared to develop more severe colitis (e.g., weight loss) than females using the DSS method, which is an observation supported by other published reports [45]. Therefore, with reports showing sex differences in AhR and colitis disease [46,47], it will be important to look more closely at how biological sex impacts these factors. Focus on addressing these aforementioned issues will be taken into consideration in future works. Collectively, the current report provides more convincing evidence that AhR ligands such as I3C are attractive candidates to be considered as preventative or therapeutic agents to use in the IBD patient population.

## 4. Materials and Methods

### 4.1. Experimental Mice

For WT animals used in this study, 8–10-week-old BALB/cJ (strain#: 000651) and C57BL/6J (strain#: 000664) mice were purchased from Jackson Laboratory (Bar Harbor, ME, USA). To generate IEC-specific conditional AhR knockout mice (AhR^ΔIEC^), Ahr^tm3.1Bra^/J (strain#: 006203) mice were crossed with B6.Cg-Tg(Vil1-cre)1000 Gum/J (strain#: 021504) mice, both of which were originally purchased from Jackson Laboratory (Bar Harbor, ME, USA). Mice were bred in-house at the animal facilities located at the University of South Carolina School of Medicine. Both breeding strains were on a C57BL/6 mouse background, and age/sex-matched WT C57BL/6 mice were used as controls. To ensure proper breeding and development of AhR^ΔIEC^, breeding strategy was based on information provided by Jackson Laboratories. However, the source of the AhR gene insert for the floxed strain (Ahr^tm3.1Bra^/J) was originally from 129SvJ mice, which carry the lower affinity *AhR^d^* allele, which were eventually crossbred into C57BL/6J mice, which carry the higher affinity *AhR^b^* allele [48]. Therefore, WT mice on C57BL/6 were used as controls. Mice were genotyped by PCR analysis of DNA isolated from tail snips using DNeasy Blood & Tissue Kit (Qiagen, Hilden, Germany) and primers designed by Jackson Laboratories and purchased from IDT Technologies (Coralville, IA, USA). To further confirm AhR cell-specific deletion in CECs, RT-PCR was performed using AhR specific mouse PrimePCR™ PreAmp mix (Bio-Rad, Hercules, CA, USA). In brief, RNA was isolated using RNeasy Micro Kit (Qiagen, Hilden, Germany) from enriched CD326^+^ (or EPCAM^+^, Biolegend, San Diego, CA, USA) cells sorted using EasySep PE Positive Selection Kit II (Stem Cell Technologies, Seattle, WA, USA) from WT, littermate (LM) and AhR^ΔIEC^ mice colons. Enrichment was confirmed by analysis on a BD FACs Celesta flow cytometer (BD Biosciences, San Jose, CA, USA). After RNA isolation, cDNA was synthesized using iscript cDNA synthesis kit (Bio-Rad) according to instructions from the manufacturer. The comparative CT (2^−ΔΔCT^) method was used to calculate fold changes for each mRNA. The mRNA levels were normalized to GAPDH, unless otherwise indicated. All mice were housed at the Association for Assessment and Accreditation of Laboratory Animal Care-accredited (AAALAC-accredited) animal facility at the University of South Carolina School of Medicine. All mice were kept in specific pathogen-free (SPF) conditions with 12 h light/dark cycles at 23 °C, 45% humidified conditions, with standard rodent chow and water available ab libitum. All PCR primers, including those used in genotyping, are provided in Table 1.

### 4.2. Induction of Colitis and I3C Administration

TNBS colitis was induced in female BALB/cJ (Jackson Laboratory, Bar Harbor, ME, USA) mice as previously described [13]. Briefly, 100 microliters containing 1 mg TNBS (5% *w*/*v*, Sigma-Aldrich, St. Louis, MO, USA) in 50% ethanol vehicle was intrarectally (i.r.) injected into mice under anesthetized conditions with 5% isoflurane. To evaluate the importance of AhR in IECs during I3C-mediated protection against colitis, WT or AhR^ΔIEC^ mice were induced with disease using the dextran sodium sulfate (DSS) method since C57BL/6J mice are more resistant to developing colitis using TNBS [49]. DSS (MP Biomedicals, Santa Ana, CA, USA) colitis was induced as previously described [13]. Briefly, male and female C57BL/6 mice were given 3% DSS (molecular weight: 36,000–50,000) in their drinking water ab libitum for 7 days, followed by regular drinking water for the remainder of the experiment. For treatment groups, I3C (40 mg/kg; Sigma-Aldrich, St. Louis, MO, USA) was dissolved in an appropriate vehicle of 0.05% dimethylsulfoxide (DMSO, Sigma-Aldrich, St. Louis, MO, USA) and corn oil (Sigma-Aldrich, St. Louis, MO, USA) and administered through the intraperitoneal (i.p.) route within 1 h of colitis induction. For the TNBS model, I3C was administered every day after colitis induction until experimental endpoint (Day 5). For the DSS model, I3C was injected every other day after colitis induction until experimental endpoint (Day 10). Dose, route, and treatment frequency of I3C were based on our previous publication showing efficacy in both the TNBS and DSS colitis models [13]. The 40 mg/kg of I3C, which is below the reported toxic levels of 250 mg/kg (i.p.) in mice [50], is the human dose equivalent (HED) to approximately 200 mg for an average 60 kg person [51]. I3C has been shown to be well tolerated in humans even at 400 mg daily [52], which is well within the range used in the current studies.

### 4.3. Assessment of Colitis Disease Severity

During all colitis experiments, mice were observed daily to include obtaining weight and evaluating stool consistency. Macroscopic colitis scores were recorded in all the experimental groups as described previously [13]. Briefly, macroscopic scores were based on three main criteria: weight loss, colon length, and stool scores. Each of the three scoring criteria ranged from 0 to 3 with a total score of 9 being the most severe score possible. Colonoscopy evaluation was also performed using a Tele Pack Vet X LED endoscope (Karl Storz, El Segundo, CA, USA) designed for small animal use. Colonoscopy scoring parameters were based on a methodology previously reported [53]. Colonoscopy scoring consisted of 4 major criteria for evaluation: perianal findings, wall transparency, intestinal bleeding, and focal lesions. Each of the four criteria were scored on a range of 0 to 3, with 12 being the maximal and most severe score. Assessment of gut permeability was also measured as described previously [13]. For gut permeability, experimental mice were administered 600 mg/kg of 4 kD fluorescein isothiocyanate (FITC)-dextran (Sigma-Aldrich, St. Louis, MO, USA) dissolved in 100 µL of phosphate-buffered solution (PBS, VWR, Radnor, PA, USA)by oral gavage; then, four hours later, blood was collected using the retroorbital method from the mice and absorbance of FITC-dextran was determined using a Multiskan FC Microplate Photometer (Thermo Fisher Scientific, Waltham, MA, USA) with an excitation wavelength of 480 nm. FITC dextran concentration was calculated using a standard curve.

### 4.4. Colon Histological Analysis

At experimental endpoints, mice were euthanized by overdose of isoflurane and colon tissues were excised. Colon tissues were flushed gently with cold phosphate-buffered solution (PBS, VWR, Radnor, PA, USA) and fixed immediately in 10% buffer-neutral formalin (VWR, Radnor, PA, USA) followed by 4% paraformaldehyde (Sigma-Aldrich, St. Louis, MO, USA) for at least 24 h prior to embedding in paraffin. Fixed colons were dehydrated by gradient alcohols, embedded in paraffin, and cut into 6 µm-thick sections. Tissue sections were then subjected to hematoxylin and eosin (H&E) staining as described previously [54]. Images of stained H&E sections were taken using a Swift M17 Trinocular LED Microscope with Integrated HD Camera (Motic, Kowloon, Hong Kong, China). Histological scoring was performed using 4 major criteria (inflammation extent, damage to crypt architecture, hyperemia/edema, infiltration with inflammatory cells) as described previously [55].

### 4.5. Evaluation of IL-22 Secretion

To evaluate IL-22 production by ILC3s, flow cytometry analysis using a BD FACs Celesta was performed on isolated cells from the lamina propria as previously described [13], using antibodies for IL-22, CD45, Lineage (Lin), CD90.2, and Rorgt (Biolegend, San Diego, CA, USA). Colonic explants were prepared as previously described with slight modifications [56]. In brief, distal colon tissues were harvested, processed, and explanted tissues were incubated at 37 °C at 5% CO_2_ for 24 h. To determine IL-22 secretion from colon explant supernatant, a mouse IL-22 enzyme-linked immunosorbent assay (ELISA) MAX Deluxe kit (Biolegend, San Diego, CA, USA) was used following instructions from the manufacturer.

### 4.6. Transcriptome Microarray Analysis of Enriched CECs

CECs were isolated from the freshly excised colon tissues as described previously [57]. Briefly, mouse colon tissues were harvested from all the experimental groups. The viability of isolated cells, determined using TC20 automated cell counter (Bio-Rad, Hercules, CA, USA), was routinely around 70–90%. After EPCAM positive cell enrichment, RNA was isolated and evaluated for microarray. Transcriptome arrays were performed using WT Pico Reagent Kit and the GCS3000 Affymetrix System (Thermo Fisher Scientific). An amount of 100 ng total RNA was used as starting material and processed as outlined in instructions from the manufacturer. Tagged samples were later hybridized to the Affymetrix Clariom D array mouse chip in Hybridization Oven 645 (Thermo Fisher Scientific, Waltham, MA, USA). Chips were washed and stained with the regents provided in the GeneChip Hybridization Wash and Stain kit (Thermo Fisher Scientific, Waltham, MA, USA) by using GeneChip Fluidics Station 450 (Thermo Fisher Scientific, Waltham, MA, USA). Chip scanning was performed by using GeneChip Scanner (Thermo Fisher Scientific) to obtain raw data. Transcriptome analysis console (TAC, Thermo Fisher Scientific), Partek Bioinformatics (Chesterfield, MO, USA), and Genesis software v 1.8.1 packages were used for downstream analysis and data visualization to generate principal coordinate analysis (PCA) plots and heatmaps.

### 4.7. Crypt Isolation and Colon Organoid Development

Colonic crypts were harvested from the colon of C57BL/6 WT and AhR^ΔIEC^ mice according to previous reports [58,59] with some modifications following Stem Cell Technologies protocols available on the manufacturer’s website. In brief, the colon was excised from euthanized experimental mice, opened longitudinally, and washed with ice-cold Ca^2+^/Mg^2+^-free sterile PBS three times. Washed colons were cut into 2–3 mm pieces with scissors and pieces were transferred to a 50 mL falcon tube, followed by further washing with cold PBS (10–15 times) with gently pipetting up and down three times. These colonic pieces were incubated in Gentle Cell Dissociation Reagent (Stem Cell Technologies, Cambridge, MA, USA) at 15–25 °C for 15 min. Cell dissociation was terminated by cold Ca^2+^/Mg^2+^-free PBS containing 0.1% BSA and crypt fractions were collected by passage through 70 μm cell strainers repeatedly and purified by centrifugation. Collections were resuspended in Advanced DMEM/F-12 (Stem Cell Technologies, Cambridge, MA, USA) after washing with PBS and counting using Rebel Hybrid Microscope Upright and Inverted Discover ECHO (VWR, Radnor, PA, USA). Collected crypts were resuspended in IntestiCult Organoid Growth Medium (Stem Cell Technologies, Cambridge, MA, USA), mixed with phenol red-free matrigel (Corning Life Sciences, Durham, NC, USA) in a 1:1 ratio, and then seeded in preheated 24-well plates. Finally, 750 μL IntestiCult Organoid Growth Medium was added to each well when matrigel solidified after incubating at 37 °C in CO_2_ incubator for 10 min. To induce injury to the colon organoids, these organoids were treated with DSS (0.03%) for 24 h after development. Previous published reports with I3C treatment in vitro with intestinal organoids use a wide range of doses, from 0.1 to 300 µM [29,60]. In initial preliminary studies using 0.1–100 µM I3C in WT-derived colon organoids, 10 µM was found to be the lowest dose able to show significant increase in Muc2 expression. Therefore, in some cases, wells were also treated with I3C (10 µM), AhR inhibitor/iAhR CH223191 (10 µM, Sigma-Aldrich, St. Louis, MO, USA), and/or mouse recombinant IL-22 (100 ng/mL, Cell Signaling Technology, Danvers, MA, USA).

### 4.8. Immunofluorescence (IF) Staining and Imaging

Organoids were grown in 4-well Nunc Lab-Tek Chamber Slide Systems (Thermo Fisher Scientific, Waltham, MA, USA) and immunostaining was performed for Muc2 as described previously [61]. In brief, organoids were fixed by removing the culture media and adding 300 μL freshly prepared 4% PFA in 10x PME buffer (500 mM PIPES, 25 mM MgCl_2_, 50 mM EDTA, Sigma-Aldrich, St. Louis, MO, USA) for 20 min at room temperature. Fixative was removed and then washed once with IF buffer (PBS containing 0.2% Triton X-100 and 0.05% Tween, Sigma-Aldrich, St. Louis, MO, USA). Organoids were permeabilized with 0.5% triton X-100 (Sigma-Aldrich, St. Louis, MO, USA) in PBS at room temperature for 30 min and incubated with a blocking buffer containing 3% bovine serum albumin (BSA, Sigma-Aldrich, St. Louis, MO, USA) in IF buffer. Organoids were stained in 1:200 dilution with an anti-Muc2 polyclonal antibody (Invitrogen, Waltham, MA, USA) in blocking solution at 4 °C overnight. Following incubation with the primary antibody, organoids were washed in 3 times for 5 min each with IF buffer and incubated with goat anti-rabbit Alexa 488 antibody (Invitrogen, Waltham, MA, USA, 1:500 in blocking solution) for 2 h and counterstained with DAPI (Invitrogen, Waltham, MA, USA, 1:1000) for 5 min. Finally, slides were mounted with ProLong™ Diamond Antifade Mount reagent (Invitrogen, Waltham, MA, USA). The images of stained organoids were captured by confocal microscopy using a Zeiss LSM 510 instrument (Leica Biosystems, Wetzlar, Germany). Confocal images were taken at 40x objective for each well, and an average of 2–3 wells were analyzed for each experimental condition. For quantification of the IF images, the ImageJ (NIH, Bethesda, MD, USA) software package v 1.54h was used. Region of Interest (ROI) manager was used to select organoid clusters to be evaluated for integrated density of both DAPI and Muc2. Within this selection, each pixel is assigned an integer that represents the fluorescent intensity from 0 to 123. The integrated density measurement, representative of fluorescent intensity, is the total sum of each pixel within the selection. Each organoid is unique in both shape and size, and to account for this variance, we used the DAPI channel as a normalization factor for each organoid. This Muc2:DAPI ratio was used to calculate fluorescence intensity.

### 4.9. In Silico Identification of DREs in Muc2 Promoter Region

Predicted AhR binding sites or DREs were identified using an online in silico tool, ConTra v3 (http://bioit2.irc.ugent.be/contra/v3, accessed on 14 February 2024). The ConTra v3 web server allows for the identification and visualization of predicted transcription factor binding sites (TFBSs) [62]. TFBS was mapped to the promoter region of the mouse Muc2 gene (NM_023566) for AhR (database: TRANSFAC20113) within 2000 base pairs using default stringency settings (core = 0.95, similarity matrix = 0.85). Similar binding sites were compared to human Muc2 region (hg18 gene; NM_002457) for potential homologous overlap in AhR binding sites between species.

### 4.10. Statistical Analysis

GraphPad Prism software version 10.1.2 (Boston, MA, USA) was used for all statistical analysis unless otherwise indicated elsewhere in the methods section. Tests and analysis were performed and conducted as previously reported [13]. When comparing 3 or more groups with one variable, one-way analysis of variance (ANOVA) and Tukey’s post hoc multiple comparisons tests were used. When comparing 3 or more groups with two variables (example: percent weight loss/gain over multiple days), two-way ANOVA and Dunnett’s post hoc multiple comparisons tests were used. For comparisons between only 2 groups, an unpaired, two-tailed standard student’s *t*-test was used. Statistical significance was determined by having a *p*-value (*p*) at least less than 0.05.

## 5. Conclusions

The current study’s findings suggest that AhR plays an important role in regulating mucin synthesis by goblet cells, notably Muc2, in both steady-state and colitis conditions. Using in vivo and in vitro approaches with WT and conditional AhR KO mice under colitis conditions, AhR was shown to affect production of Muc2 after stimulation with an AhR ligand (I3C), and this appeared to be independent of IL-22. Based on the results, IL-22 likely affects goblet cell differentiation and development, not mucus production, which is more likely regulated directly by AhR, which needs to be further confirmed in future studies.

## Figures and Tables

**Table 1:** Primers for PCR.

Primer	Forward	Reverse
AhR	CAGTGGGAATAAGGCAAGAGTGA	GGTACAAGTGCACATGCCTGC
Vil1	GCCTTCTCCTCTAGGCTCGT	AGGCAAATTTTGGTGTACGG
Vil1 IP	-	TATAGGGCAGAGCTGGAGGA
GAPDH	AACAGCAACTCCCACTCTTC	CCTGTTGCTGTAGCCGTATT
Muc1	TGGATTGTTTCTGCAGATTTT	CCTGACCTGAACTTGATGCT
Muc2	CTACCATTACCACCACTAC	GTCTCTCGATCACCACCATTT
Muc3	TGTTCAGCTTTACTGTGTTTCAA	TTGCATGTCTCCTCAGGATT
Tff3	TAATGCTGTTGGTGGTCCTG	CAGCCACGGTTGTTACACTG

## Data Availability

The raw microarray data supporting the conclusions of this article can be found deposited at the NCBI Gene Expression Omnibus public database with accession numbers GSE242890 and GSE242891. Any other raw data supporting the conclusions of this article will be made available by the authors, without undue reservation.

## Supplementary Materials

The following supporting information can be downloaded at: https://www.mdpi.com/article/10.3390/ijms25042404/s1.
