# Supplementary material for: Aryl Hydrocarbon Receptor Regulates Muc2 Production Independently of IL-22 during Colitis
Source: Int J Mol Sci. 2024 Feb 18;25(4):2404. doi: 10.3390/ijms25042404 (PMC10888603; doi:10.3390/ijms25042404)
Supplement: Supplementary file 1 [file ijms-25-02404-s001.zip › ijms-2843281-supplementary.pdf]

Supplementation information

Aryl hydrocarbon receptor regulates Muc2 production independent of IL-22 during colitis

Archana Saxena, Chandani Mitchell, Raymond Bogdon, Kasie Roark, Kiesha Wilson, Shanieka Staley, Michelle Hailey, Michal Claire Williams, Alex Rutkovsky, Prakash Nagarkatti, Mitzi Nagarkatti, and Philip Brandon Busbee

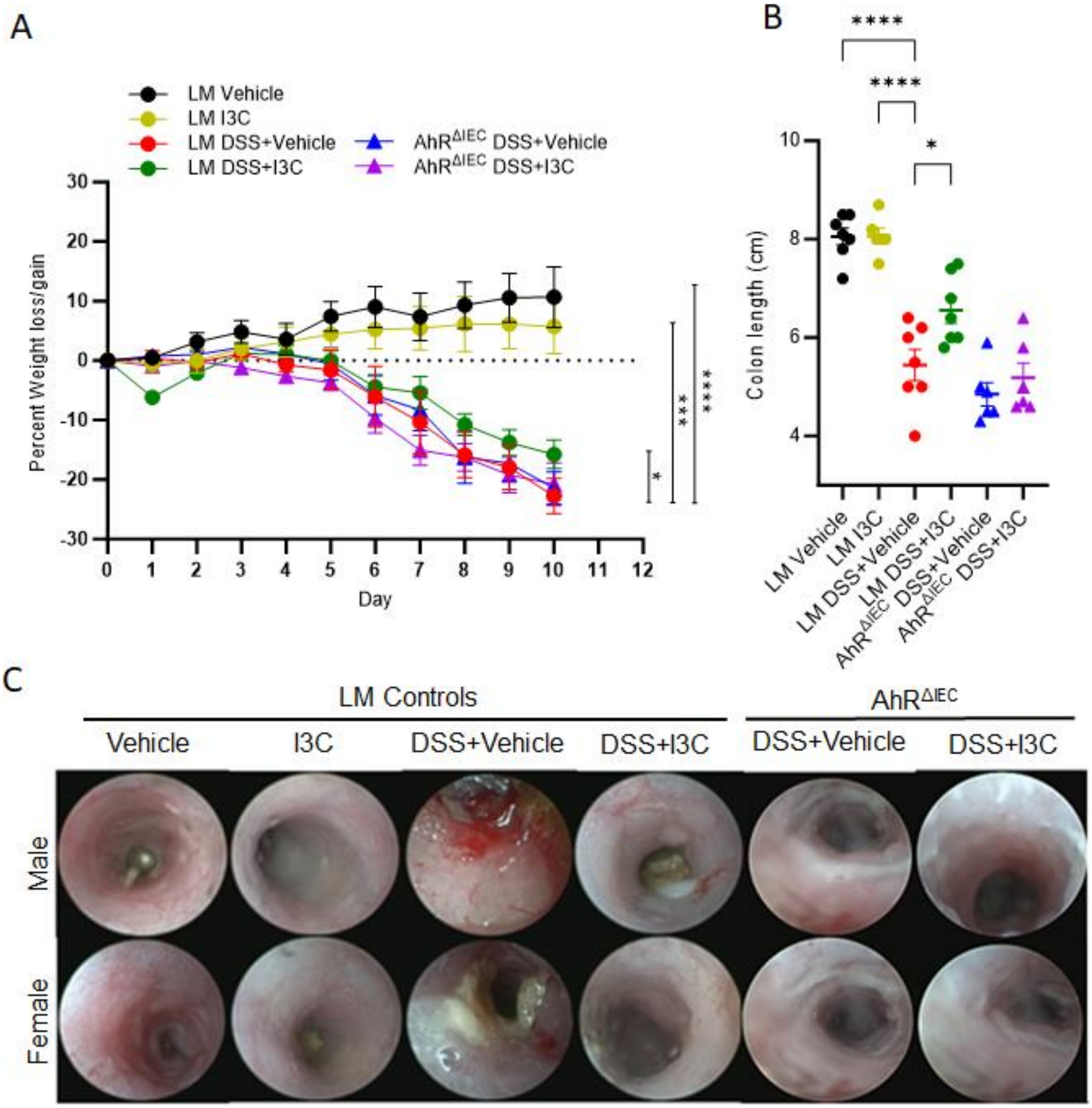

Figure S1. AhR expression in IECs is essential for I3C-mediated protection against DSS-induced colitis in littermate (LM) male and female mice, related to Figures 1-3. (**A**) Percent weight data. (**B**) Colon length. (**C**) Representative colonoscopy images taken on day 9 of DSS colitis from experimental groups. For dot plots, significance was determined using one-way ANOVA and Tukey's multiple comparisons test. For weight data over time, two-way ANOVA and Dunnett's multiple comparisons test was used to determine significance (*p<0.05, ***p<0.005, ****p<0.001).

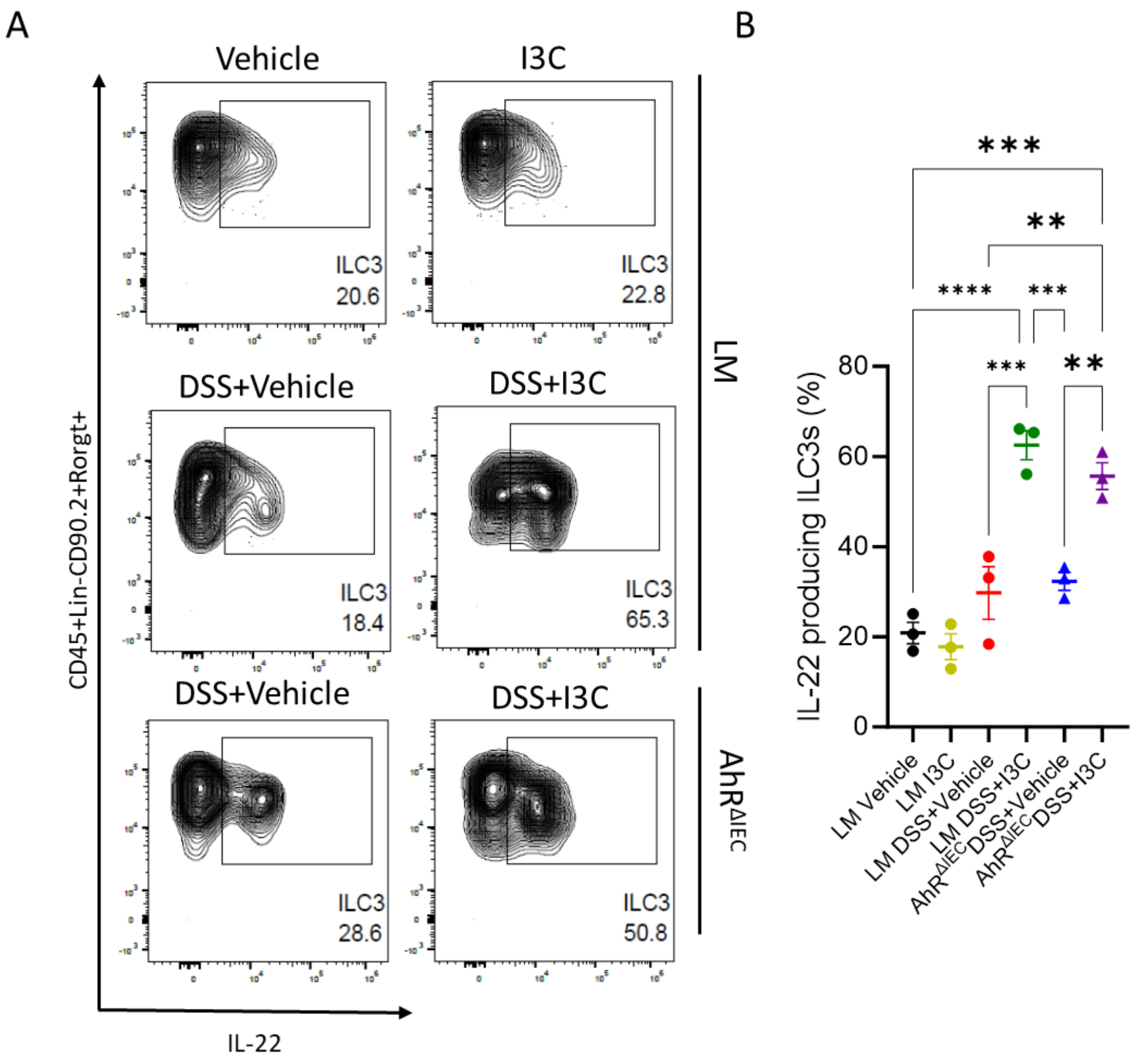

Figure S2. AhR expression in IECs does not impact production of IL-22 by ILC3s during DSS-induced colitis and treatment with I3C, related to Figures 1-3. (**A**) Representative flow cytometry plots depicting percentage of IL-22 producing ILC3s (CD45+Lin-CD90.2+Rorgt+). (**B**) Dot plot representing percent IL-22-producing ILC3s from experimental groups. For dot plots, significance was determined using one-way ANOVA and Tukey's multiple comparisons test. For weight data over time, two-way ANOVA and Dunnett's multiple comparisons test was used to determine significance (**p<0.01, ***p<0.005, ****p<0.001).
